# *In silico* evaluation of soursop (*Annona muricata*) leaf compound interactions with CYP450 and their potential impact on systemic cancer therapies

**DOI:** 10.1016/j.toxrep.2026.102258

**Published:** 2026-04-19

**Authors:** Ricardo Ballesteros-Ramírez, Daniel Fuenmayor, Nicolás Martínez-Ramos, Eduardo Duque-Grisales, Raúl Murillo

**Affiliations:** a Grupo de Oncología PUJ-HUSI. Facultad de Medicina, Pontificia Universidad Javeriana, Bogotá, Colombia; b Centro Javeriano de Oncología, Hospital Universitario San Ignacio, Bogotá, DC, Colombia; c Semillero de Oncología, Facultad de Medicina, Pontificia Universidad Javeriana, Bogotá, DC, Colombia; d Institución Universitaria Esumer, Facultad de Estudios Empresariales, Medellín, Colombia; e Institución Universitario Pascual Bravo, Facultad de Ingeniería, Medellín, Colombia

**Keywords:** Annona muricata, CYP450, Herb-drug interactions, In silico modeling, ADME, Pharmacokinetics, Cancer therapy

## Abstract

*Annona muricata* (soursop, graviola) is a tropical plant widely used in traditional medicine for the management of various ailments, including cancer. Its leaf and fruit extracts are frequently consumed as complementary therapies by oncology patients, yet their potential to interfere with CYP450-mediated drug metabolism remains poorly characterized. This study employed two complementary in silico platforms, SuperCYPsPred for CYP450 interaction probability predictions and SwissADME for ADME/pharmacokinetic profiling, to evaluate 12 representative leaf compounds, as leaves constitute the most used plant part in traditional medicinal preparations of *A. muricata*. SuperCYPsPred analysis identified aporphine alkaloids as having the highest predicted interaction probabilities: isolaureline (mean 61.5%), xylopine (59.9%), and anonaine (55.9%), with particularly high CYP2D6-specific probabilities (94.9%, 88.8%, and 85.7%, respectively). SwissADME analysis revealed that these same compounds exhibit high gastrointestinal absorption, blood-brain barrier permeability, favorable drug-likeness, and predicted CYP2D6 inhibition. Integrated risk assessment combining both platforms identified four high-risk compounds (isolaureline, xylopine, anonaine, stepharine) and two moderate-risk compounds (coclaurine, reticuline) that may warrant clinical monitoring during concurrent chemotherapy. Conversely, acetogenins showed low oral bioavailability and minimal CYP interaction potential, suggesting limited systemic exposure. These findings suggest that specific alkaloid classes in soursop preparations may pose a greater pharmacokinetic interaction risk than previously recognized, particularly for drugs metabolized by CYP2D6. Further experimental validation is warranted to ensure the safe use of *A. muricata* preparations in oncology care.

## Highlights

• Dual *in silico* approach assessed CYP450 interaction risk of *A. muricata* compounds.
• Aporphine alkaloids showed highest predicted CYP2D6 probabilities (85–95%).
• SuperCYPsPred and SwissADME independently confirmed CYP2D6 as primary target.
• Acetogenins showed low oral bioavailability, mitigating herb–drug interaction risk.
• Four HIGH-risk compounds identified for CYP2D6-metabolized cancer therapies.

## Graphical Abstract

**Figure:**
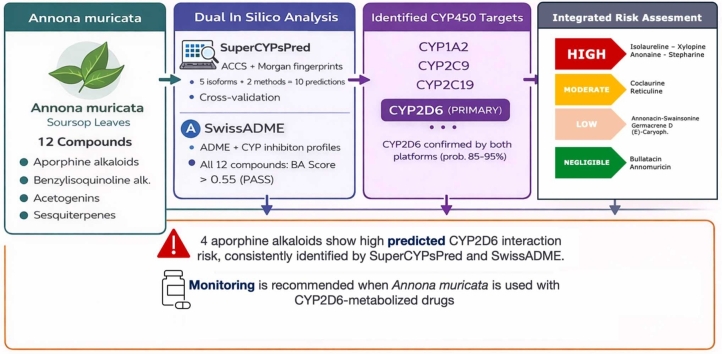

## 1 Introduction

*Annona muricata* L. (Annonaceae), commonly known as soursop or guanábana, is a tropical tree whose leaves have been widely used in traditional medicine across Latin America, Africa, and Southeast Asia [1]. In oncology settings, soursop leaf preparations are increasingly consumed by cancer patients as complementary therapy, often without disclosure to their treating physicians [2]. Our group previously conducted a large-scale survey involving over 3000 oncology patients in Colombia and identified soursop leaf preparations as the most frequently used herbal product [3]. Regional variability in herbal preparation methods (decoction, infusion, tincture) and dosages across different cultural traditions may influence the concentration of bioactive compounds reaching systemic circulation. This self-medication raises concerns about potential pharmacokinetic interactions between soursop bioactive compounds and conventional chemotherapeutic agents [4].

The cytochrome P450 (CYP450) enzyme superfamily mediates the metabolism of approximately 75% of clinically used drugs [5]. Five major isoforms (CYP1A2, CYP2C9, CYP2C19, CYP2D6, and CYP3A4) metabolize most chemotherapeutic agents, including tamoxifen (CYP2D6), erlotinib (CYP1A2/CYP3A4), docetaxel (CYP3A4), and irinotecan (CYP3A4/CYP2C19). Herb-drug interactions mediated through CYP450 modulation represent a recognized clinical concern [6].

Previous phytochemical analyses have identified over 200 bioactive compounds in *A. muricata* leaves, including acetogenins, aporphine alkaloids, benzylisoquinoline alkaloids, and terpenes [7]. Bioactive compound profiling in related Annonaceae species has further confirmed the presence of acetogenins with significant biological activities [8]. While several studies have investigated the cytotoxic properties of these compounds, systematic evaluation of their CYP450 interaction potential combined with pharmacokinetic profiling remains absent from the literature.

This report employed two complementary *in silico* approaches: (1) SuperCYPsPred, a validated machine-learning platform for predicting CYP450 interaction probabilities, and (2) SwissADME, a web-based tool for evaluating ADME properties and drug-likeness. By integrating CYP450 interaction predictions with pharmacokinetic profiling, this study provides a clinically relevant *in silico* risk assessment framework for *A. muricata* compounds during concurrent cancer pharmacotherapy, laying the groundwork for safer integrative oncology practices and future experimental validation.

## 2 Materials and methods

### 2.1 Compound selection

A literature review was conducted to identify phytochemical constituents of *Annona muricata*, focusing on studies published between 2007 and 2025. Data from various plant parts (leaves, seeds, pulp, bark, and roots) were compiled, with a specific focus on compounds present in the leaves due to their prevalent use in cancer patients. Twelve representative compounds from *A. muricata* leaves were selected based on published phytochemical analyses: anonaine, xylopine, isolaureline (aporphine alkaloids); coclaurine, reticuline (benzylisoquinoline alkaloids); stepharine (proaporphine); swainsonine (indolizidine alkaloid); annonacin, bullatacin, annomuricin (acetogenins); and germacrene D, (*E*)-caryophyllene (sesquiterpenes). Canonical SMILES were obtained from PubChem [9]. Table S1 was used to support compound selection and prioritization.

### 2.2 CYP450 interaction prediction (SuperCYPsPred) and ADME/pharmacokinetic profiling (SwissADME)

CYP450 interaction predictions were performed using SuperCYPsPred (https://prediction.charite.de/subpages/supercypspred.php), a validated machine-learning platform [10]. The platform evaluates interaction probabilities across five CYP isoforms (CYP1A2, CYP2C9, CYP2C19, CYP2D6, CYP3A4) using two independent molecular fingerprint methods, MACCS keys and Morgan circular fingerprints, yielding 10 CYP isoform-method combinations per compound (5 isoforms × 2 methods). Default probability thresholds were applied: compounds with predicted probability > 0.5 were classified as “Active” (predicted interaction), while those ≤ 0.5 were classified as “Inactive.” It is important to note that SuperCYPsPred provides probabilistic interaction predictions and does not distinguish between inhibition, induction, or substrate behavior.

To validate the platform’s predictive utility, benchmark analyses were conducted using five well-characterized pharmaceutical CYP inhibitors: ketoconazole (CYP3A4, correctly predicted Active at 100%/99.0% MACCS/Morgan), quinidine (CYP2D6, Active at 98.2%/98.7%), fluvoxamine (CYP1A2, Active at 83.7%/83.1%), fluconazole (CYP2C9, Active at 52.5% via MACCS), and omeprazole (CYP2C19, Active at 99.1%/70.4%). All primary CYP targets were correctly identified. This constitutes a qualitative directional validation; formal sensitivity/specificity metrics and false positive rates were not calculated, as the benchmark set was limited to five reference compounds intended to confirm predictive directionality rather than to provide comprehensive external validation. Additionally, three well-characterized dietary CYP modulators (naringenin, quercetin, and bergamottin) were evaluated as comparative context.

Pharmacokinetic properties were evaluated using SwissADME (http://www.swissadme.ch/) [11]. Each compound’s canonical SMILES was individually submitted, and the following parameters were recorded: molecular weight, consensus Log P, TPSA, predicted GI absorption, BBB permeability, CYP inhibition predictions, Lipinski violations, and bioavailability score (BA Score). Compounds with BA Score > 0.30 were considered to have sufficient predicted oral bioavailability for systemic pharmacokinetic relevance [12].

### 2.3 Integrated risk assessment

An integrated risk assessment framework was developed combining SuperCYPsPred interaction probabilities with SwissADME pharmacokinetic parameters. Compounds were classified into four risk categories based on the convergence of evidence: HIGH risk (SuperCYPsPred mean probability > 50% combined with High GI absorption, CYP inhibition confirmed by SwissADME, and zero Lipinski violations); MODERATE risk (mean probability 30–50% with High GI absorption and CYP inhibition confirmed); LOW risk (mean probability < 30% or Low GI absorption); and NEGLIGIBLE risk (low probability combined with low GI absorption and no CYP inhibition by SwissADME). This dual-platform approach leverages the complementary strengths of both tools; however, this risk stratification framework remains exploratory and has not been clinically validated. The proposed categories are intended to prioritize compounds for future experimental investigation rather than to establish definitive clinical risk levels.

## 3 Results

**Fig. 1:**
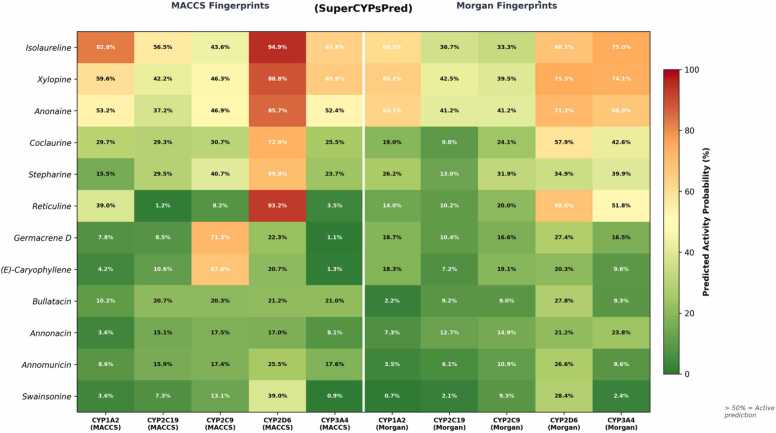
Heatmap of predicted CYP450 interaction probabilities for twelve *A. muricata* compounds across 10 CYP isoform-method combinations (SuperCYPsPred). Darker red indicates higher predicted interaction probability.

**Fig. 2:**
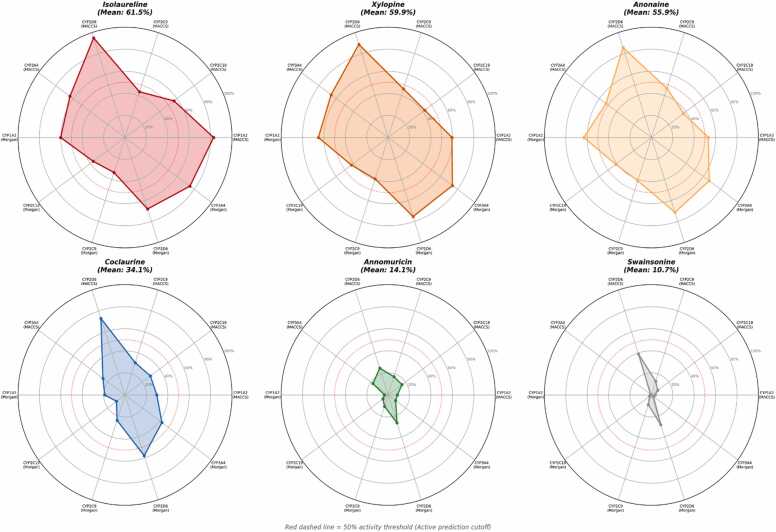
Radar plots of CYP450 interaction profiles for six representative compounds. Top row: high-interaction compounds (isolaureline, xylopine, anonaine); bottom row: low-interaction comparators (swainsonine, annonacin, (*E*)-caryophyllene). Dashed line indicates the 50% probability threshold.

SuperCYPsPred analysis of twelve compounds across 10 CYP isoform-method combinations revealed a clear structure-activity pattern (Fig. 1, Table S2). Aporphine alkaloids exhibited the highest mean predicted interaction probabilities: isolaureline (61.5%), xylopine (59.9%), and anonaine (55.9%), with particularly high CYP2D6-specific probabilities (94.9%, 88.8%, and 85.7% via MACCS fingerprints, respectively). Benzylisoquinoline alkaloids showed intermediate probabilities: coclaurine (34.1%) and reticuline (31.0%), with the highest predictions also directed toward CYP2D6. Stepharine (32.5%) showed a distinctive broad-spectrum profile across CYP1A2, CYP2C19, CYP2D6, and CYP3A4. In contrast, acetogenins (14.1–15.1%), terpenes (17.9–20.1%), and swainsonine (10.7%) exhibited low interaction probabilities with predominantly Inactive classifications (Fig. 2).

Benchmark validation confirmed that SuperCYPsPred correctly identified primary CYP targets for all five reference inhibitors. Comparison with dietary CYP modulators showed that naringenin (6/10 active predictions), quercetin (5/10), and bergamottin (8/10) had broad multi-CYP profiles, whereas the *A. muricata* alkaloids showed fewer total active predictions but notably higher CYP2D6-specific probabilities.

SwissADME analysis revealed striking pharmacokinetic differences between compound classes (Table 1). All twelve compounds passed the OB > 0.30 threshold (BA Score = 0.55). Alkaloids consistently demonstrated high predicted GI absorption, BBB permeability (except swainsonine), and zero Lipinski violations (MW 173–329 g/mol, cLogP –0.44–3.71). In contrast, acetogenins displayed poor drug-likeness: one Lipinski violation each, MW > 500 g/mol, high lipophilicity (Log P 6.57–8.24), low GI absorption, no BBB permeation, and high TPSA (96.2–119.6 Å²). Sesquiterpenes showed low GI absorption despite small molecular size, attributable to their purely hydrocarbon nature (TPSA = 0.00 Å²).

**Table 1:** ADME/pharmacokinetic properties of twelve *A. muricata* compounds (SwissADME). GI = gastrointestinal absorption; BBB = blood-brain barrier; BA = bioavailability score; TPSA = topological polar surface area.

**Compound**	**MW (g/mol)**	**cLogP**	**GI Abs.**	**BBB**	**Lipinski Viol.**	**BA Score**	**SwissADME CYP Inhib.**	**TPSA (Å²)**
Anonaine	265.3	3.71	High	Yes	0	0.55	1A2, 2D6	31.4
Xylopine	295.3	2.62	High	Yes	0	0.55	1A2, 2D6	40.6
Isolaureline	309.4	3.28	High	Yes	0	0.55	1A2, 2D6	40.6
Coclaurine	285.3	2.14	High	Yes	0	0.55	2D6	63.9
Reticuline	329.4	2.60	High	Yes	0	0.55	2D6	62.2
Stepharine	323.4	2.69	High	Yes	0	0.55	1A2, 2C19, 2D6, 3A4	41.9
Swainsonine	173.2	–0.44	High	No	0	0.55	None	63.9
Annonacin	552.8	7.15	Low	No	1	0.55	3A4	96.2
Bullatacin	600.9	6.57	Low	No	1	0.55	None	119.6
Annomuricin	613.0	8.24	Low	No	1	0.55	None	99.4
Germacrene D	218.4	4.88	Low	No	1	0.55	2C9	0.0
(*E*)-Caryoph.	162.3	3.27	Low	Yes	0	0.55	None	0.0

CYP inhibition predictions from SwissADME showed notable concordance with SuperCYPsPred results. All three aporphine alkaloids were predicted as CYP1A2 and CYP2D6 inhibitors by SwissADME, consistent with their high SuperCYPsPred probabilities. Stepharine was predicted to inhibit four of five CYPs (CYP1A2, CYP2C19, CYP2D6, CYP3A4), making it the broadest CYP inhibitor in this dataset. Swainsonine was predicted not to inhibit any CYP isoform, consistent with its low SuperCYPsPred probability (mean 10.7%).

Integration of SuperCYPsPred interaction probabilities with SwissADME pharmacokinetic parameters yielded a stratified risk assessment (Table 2). Four compounds were classified as HIGH risk: isolaureline, xylopine, anonaine, and stepharine. These share the critical combination of high predicted CYP interaction probabilities, high GI absorption, BBB permeability, CYP inhibition confirmed independently by SwissADME, and zero Lipinski violations. Two compounds (coclaurine, reticuline) were classified as MODERATE risk based on lower SuperCYPsPred probabilities (31–34%) but confirmed CYP2D6 inhibition and high GI absorption. The remaining six compounds were classified as LOW or NEGLIGIBLE risk, primarily due to low GI absorption (acetogenins, terpenes) or absence of CYP inhibition (swainsonine, bullatacin, annomuricin, (*E*)-caryophyllene).

**Table 2:** Integrated risk assessment combining SuperCYPsPred CYP450 interaction predictions with SwissADME ADME/pharmacokinetic profiling. Risk levels reflect the convergence of predicted interaction probability, oral bioavailability, and drug-likeness.

**Compound**	**SuperCYPsPred Mean (%)**	**SwissADME CYP Inhib.**	**GI Abs.**	**BBB**	**Lipinski (Viol.)**	**Integrated Risk Level**
Isolaureline	61.5	1A2, 2D6	High	Yes	Pass (0)	**HIGH**
Xylopine	59.9	1A2, 2D6	High	Yes	Pass (0)	**HIGH**
Anonaine	55.9	1A2, 2D6	High	Yes	Pass (0)	**HIGH**
Stepharine	32.5	1A2, 2C19, 2D6, 3A4	High	Yes	Pass (0)	**HIGH**
Coclaurine	34.1	2D6	High	Yes	Pass (0)	**MODERATE**
Reticuline	31.0	2D6	High	Yes	Pass (0)	**MODERATE**
Annonacin	14.1	3A4	Low	No	1 viol.	**LOW**
Swainsonine	10.7	None	High	No	Pass (0)	**LOW**
Germacrene D	20.1	2C9	Low	No	1 viol.	**LOW**
(*E*)-Caryoph.	17.9	None	Low	Yes	Pass (0)	**LOW**
Bullatacin	15.1	None	Low	No	1 viol.	**NEGLIGIBLE**
Annomuricin	14.1	None	Low	No	1 viol.	**NEGLIGIBLE**

## 4 Discussion

This study represents the integrated *in silico* assessment combining CYP450 interaction predictions (SuperCYPsPred) with ADME profiling (SwissADME) for *A. muricata* leaf compounds. The most significant finding is the convergent high-risk profile of aporphine alkaloids (isolaureline, xylopine, anonaine). These compounds demonstrate: (i) the highest SuperCYPsPred interaction probabilities across the dataset, specifically targeting CYP2D6 (85–95% via MACCS); (ii) high GI absorption and BBB permeability; (iii) favorable drug-likeness with zero Lipinski violations; and (iv) independent confirmation of CYP1A2 and CYP2D6 inhibition by SwissADME. This convergence from two independent platforms increases confidence in the predicted CYP interaction profiles of these compounds. However, it must be noted that neither platform models concentration-dependent inhibition, binding kinetics, or enzyme turnover. Predicted GI absorption indicates favorable physicochemical properties for oral absorption but does not demonstrate that pharmacologically relevant systemic concentrations are achieved following herbal consumption.

CYP2D6 metabolizes approximately 25% of clinically used drugs and exhibits well-documented polymorphisms across populations [13], [14]. Poor metabolizers, who represent 5–10% of Caucasian and 1–2% of Asian populations, may be particularly susceptible to further CYP2D6 inhibition by exogenous compounds. For example, tamoxifen, a first-line hormonal therapy for estrogen receptor-positive breast cancer, requires CYP2D6-mediated bioactivation to its active metabolite endoxifen, co-administration with CYP2D6 inhibitors may reduce therapeutic efficacy [15]. However, this potential interaction remains hypothetical in the absence of in vitro enzyme inhibition assays, metabolic flux analysis, or pharmacokinetic simulations specific to *A. muricata* compounds.

Stepharine warrants particular attention due to its predicted inhibition of four of five major CYP isoforms (CYP1A2, CYP2C19, CYP2D6, CYP3A4) by both platforms. While its mean SuperCYPsPred probability (32.5%) is lower than the aporphines, the breadth of its predicted CYP interactions, combined with high GI absorption and BBB permeability, suggests potential for polypharmacy interactions across multiple drug classes. This profile is particularly concerning for cancer patients receiving multi-drug regimens.

A particularly important result is that the acetogenins (annonacin, bullatacin, annomuricin), despite being the most studied *A. muricata* compounds for their anticancer properties, showed low predicted oral bioavailability (Low GI absorption, One Lipinski violation each, Log P > 6.5). This substantially mitigates their herb-drug interaction risk via the oral route. Comparison with known dietary CYP modulators provides important context. Naringenin (grapefruit flavonoid, 6/10 active SuperCYPsPred predictions), quercetin (5/10 active), and bergamottin (grapefruit furanocoumarin, 8/10 active) showed broad multi-CYP interaction profiles. The top *A. muricata* alkaloids showed fewer total active predictions but notably higher CYP2D6-specific probabilities, suggesting a more targeted but potentially more clinically significant interaction profile for CYP2D6-metabolized drugs. The preferential CYP2D6 selectivity of aporphine alkaloids may relate to their rigid planar tetracyclic ring system containing a protonatable nitrogen, which is consistent with the restricted active site cavity of CYP2D6 that favors compact, lipophilic bases [5], [16]. This differs structurally from the broader flavone scaffold of dietary modulators, whose multiple hydroxyl groups allow interactions across several CYP active sites. However, direct probability comparisons should be interpreted cautiously, as relative inhibitory potency cannot be inferred from predicted interaction probability alone without concentration-response data.

The concordance between SuperCYPsPred and SwissADME CYP inhibition predictions is notable given that the two tools employ fundamentally different computational approaches: machine-learning classifiers trained on known CYP modulators versus support vector machine (SVM) models for CYP inhibition prediction. This methodological independence lends greater weight to the convergent identification of CYP2D6 as the primary predicted target for aporphine alkaloids.

Several limitations must be acknowledged. First, both SuperCYPsPred and SwissADME provide *in silico* predictions based on molecular structure; no direct drug-compound interaction was modeled in this study. Second, estimated plasma concentrations of these alkaloids following typical herbal preparation consumption remain unknown, representing a critical gap that limits translational interpretation. It should be noted that SwissADME predictions of GI absorption and drug-likeness do not substitute for experimental pharmacokinetic data; systemic exposure from herbal preparations remains unknown, and the risk classification should not be interpreted as implying demonstrated pharmacokinetic relevance. Third, the integrated risk classification, while based on objective parameters from two independent platforms, has not been validated against clinical outcome data; accordingly, the proposed risk categories should be regarded as exploratory and hypothesis-generating. Fourth, synergistic or antagonistic effects between co-occurring compounds in crude soursop extracts were not evaluated. Fifth, regional variability in herbal preparation methods and phytochemical composition may substantially influence actual compound exposure.

## 5 Conclusions

Integrated *in silico* analysis combining SuperCYPsPred and SwissADME identified a subset of *A. muricata* alkaloids that may pose a pharmacokinetic interaction risk with concurrent cancer pharmacotherapy. Isolaureline, xylopine, and anonaine (aporphine alkaloids) represent the highest-risk compounds. Stepharine additionally shows broad-spectrum CYP inhibition across four isoforms. Acetogenins (annonacin, bullatacin, annomuricin), despite their well-documented cytotoxic properties, are predicted to have low oral bioavailability and minimal CYP interaction potential. Swainsonine shows consistently low CYP interaction probability and no CYP inhibition by SwissADME. These results, derived from an exploratory in silico framework that has not been clinically validated, suggest that healthcare providers should consider the potential for pharmacokinetic interactions when counseling cancer patients who consume*A. muricata* preparations, particularly those receiving CYP2D6-metabolized therapies (tamoxifen, codeine, ondansetron). *In vitro* validation using human liver microsomes or recombinant CYP assays, along with pharmacokinetic studies determining actual plasma concentrations of these alkaloids after herbal consumption, is recommended as the next translational step.

## CRediT authorship contribution statement

**Ricardo Ballesteros-Ramírez:** Writing – review & editing, Writing – original draft, Project administration, Methodology, Formal analysis, Conceptualization. **Daniel Fuenmayor:** Methodology, Formal analysis, Data curation, Conceptualization. **Nicolás Martínez-Ramos:** Writing – original draft, Methodology, Formal analysis, Data curation. **Eduardo Duque-Grisales:** Methodology, Formal analysis. **Raúl Murillo:** Writing – review & editing, Methodology, Investigation, Funding acquisition, Formal analysis, Conceptualization.

## Funding

The authors acknowledge financial support for the publication of this article from the Vicerrectoría de Investigación of the 10.13039/501100009543Pontificia Universidad Javeriana.

## Declaration of Competing Interest

The authors declare that they have no known competing financial interests or personal relationships that could have appeared to influence the work reported in this paper.

## Data Availability

Data will be made available on request.
