# Supplementary material for: In silico evaluation of soursop (Annona muricata) leaf compound interactions with CYP450 and their potential impact on systemic cancer therapies
Source: Toxicol Rep. 2026 Apr 19;16:102258. doi: 10.1016/j.toxrep.2026.102258 (PMC13140062; doi:10.1016/j.toxrep.2026.102258)
Supplement: Supplementary file 1 — Supplementary material [file mmc1.docx]

**Table S1.** Literature Supporting Compound Selection for *In Silico* CYP450 Interaction Analysis of *Annona muricata* Leaf Compounds

| **#** | **Reference** | **Year** | **Plant Part** | **Compound Classes Identified** | **Main Phytochemical Findings** | **Relevance to This Study** |
| --- | --- | --- | --- | --- | --- | --- |
| 1 | Kossouoh C et al. | 2007 | Leaves | Terpenoids, essential oils | Characterized essential oil composition of A. muricata leaves from Benin, identifying sesquiterpenes as major volatile constituents | Supports the inclusion of germacrene D and (E)-caryophyllene as representative sesquiterpenes in our CYP450 interaction analysis. Both showed low predicted interaction probabilities (20.1% and 17.9%) and low GI absorption. |
| 2 | Lako J et al. | 2007 | Pulp | Flavonols, carotenoids, polyphenols | Quantified flavonol and carotenoid content in Fijian A. muricata pulp with antioxidant assessment | Documents the polyphenolic diversity in soursop, although pulp-specific flavonols were not prioritized in our leaf-focused CYP interaction analysis. |
| 3 | Márques V & Farah A | 2009 | Leaves | Chlorogenic acids, phenolic compounds | Identified chlorogenic acid derivatives and other phenolics in A. muricata leaf infusions | Confirms the presence of phenolic compounds in leaf preparations; these may contribute to antioxidant but not CYP-mediated interactions based on current evidence. |
| 4 | Hassimotto NMA et al. | 2009 | Frozen pulp | Phenolic acids, ascorbic acid | Measured antioxidant capacity and phenolic acid profiles in commercially available soursop frozen pulp | Provides baseline data on phenolic content in processed soursop products relevant to consumer exposure assessment. |
| 5 | Isabelle M et al. | 2010 | Pulp | Phenolics, vitamin C | Profiled antioxidant activity and phenolic/vitamin C content in soursop pulp from Singapore | Contributes to understanding regional variation in phytochemical composition, a factor acknowledged as a limitation in our study. |
| 6 | Ogunlesi M et al. | 2010 | Pulp | Ascorbic acid (vitamin C) | Determined vitamin C content in tropical fruits including A. muricata using voltammetric methods | Documents nutritional composition; vitamin C is not a known CYP modulator and was therefore not included in our interaction analysis. |
| 7 | Ortiz D et al. | 2012 | Various parts | Phenolics, flavonoids | Comprehensive bibliographic review of antioxidant activity in A. muricata across plant tissues | Provided foundational knowledge on the distribution of phenolic and flavonoid compounds across different plant parts of soursop. |
| 8 | Vijayameena C et al. | 2013 | Pulp, seeds, leaves | Alkaloids, tannins, flavonoids | Phytochemical screening confirmed presence of alkaloids, tannins, and flavonoids with antibacterial activity in leaf and seed extracts | Supports the selection of alkaloids as a key compound class. Alkaloids (anonaine, xylopine, isolaureline, coclaurine, reticuline, stepharine) showed the highest CYP interaction probabilities in our analysis (31–62%). |
| 9 | Thang TD et al. | 2013 | Leaves | Sesquiterpenes, monoterpenes (volatile oils) | Characterized volatile oil composition in Annona species from Vietnam, identifying β-caryophyllene and germacrene D among major constituents | Directly supports the inclusion of (E)-caryophyllene and germacrene D as representative sesquiterpenes. Both showed low CYP interaction risk (LOW category) in our integrated assessment. |
| 10 | Owolabi MS et al. | 2013 | Leaves | Essential oils, acetogenins | Analyzed leaf essential oil composition from Badagary, Nigeria and reported cytotoxic activity associated with annonacin | Confirms annonacin as a key bioactive acetogenin in leaves. Our analysis showed annonacin has low GI absorption but predicted CYP3A4 inhibition (SwissADME), classified as LOW risk. |
| 11 | Stephen A | 2015 | Peel, pulp, seed | Phenolics, flavonoids | Evaluated phenolic distribution, antidiabetic, and antihypertensive properties across soursop fruit tissues | Documents the pharmacological activity of soursop phenolics across tissues; supports the broader clinical context of herb-drug interaction potential in patients using soursop for multiple conditions. |
| 12 | Kingsley C et al. | 2017 | Various parts | Alkaloids, phenolics, flavonoids | Proximate composition and phytochemical analysis with quantification of alkaloid, phenolic, and flavonoid content and antioxidant potential | Confirms the co-occurrence of alkaloids and phenolics in crude extracts, supporting the relevance of evaluating multiple compound classes for CYP interactions. |
| 13 | Benatti Justino A et al. | 2018 | Leaves | Polyphenols, flavonoids, glycosides | Identified A. muricata leaf polyphenols as sources of antioxidant and antidiabetic compounds with in vitro α-glucosidase inhibition | Highlights additional pharmacological activities of leaf preparations that may lead to concurrent use with antidiabetic medications, increasing the clinical relevance of CYP interaction assessment. |
| 14 | Nugraha AS et al. | 2019 | Root | Alkaloids (muricin, reticuline) | Isolated and characterized alkaloids from Indonesian A. muricata roots, including reticuline | Directly supports reticuline as a confirmed A. muricata alkaloid. In our analysis, reticuline showed MODERATE CYP interaction risk with predicted CYP2D6 inhibition (31.0% SuperCYPsPred, confirmed by SwissADME). |
| 15 | Nugraha AS et al. | 2019 | Root | Alkaloids, acetogenins | Reviewed anti-infective and anticancer properties of Annona species with focus on ethnomedicinal uses and isolated compounds | Comprehensive review supporting compound selection; confirms the pharmacological significance of both alkaloid and acetogenin classes evaluated in our CYP450 study. |
| 16 | Taiwo F | 2019 | Leaves | Kaempferol-3-O-glucoside, phenolics | Isolated kaempferol-3-O-glucoside from leaves and demonstrated antimicrobial and antioxidant properties | Confirms flavonoid glycosides in leaves; while kaempferol derivatives were not among our 12 selected compounds, their presence highlights the chemical complexity of crude leaf extracts. |
| 17 | Valdez-Guerrero et al. | 2019 | Leaves | Polyphenols, flavonoids | Developed green chemistry extraction protocols for polyphenols from A. muricata leaves | Demonstrates that extraction methodology significantly affects phytochemical yield; relevant to our discussion of regional and methodological variability in preparation methods. |
| 18 | Jocelin Chan W-J et al. | 2020 | Leaves | Safety/toxicity evaluation | Systematic review evaluating the safety and tolerability of A. muricata leaf extracts in preclinical and clinical settings | Critical reference for the safety context of our study. Supports the need for CYP interaction assessment as part of a comprehensive safety evaluation of soursop leaf preparations. |
| 19 | Nguyen MT et al. | 2020 | Leaves | Polyphenols, flavonoids | Quantified total polyphenol and flavonoid content with antioxidant activity assessment using DPPH and ABTS assays | Provides quantitative phytochemical data supporting the presence of bioactive compounds in leaf preparations consumed by patients. |
| 20 | Aguilar-Hernández G et al. | 2020 | Pulp, peel, seed | Alkaloids, acetogenins | Developed ultrasound-assisted extraction protocols for alkaloids from soursop fruit pulp and by-products | Confirms alkaloid presence in multiple plant parts beyond leaves; relevant to understanding total alkaloid exposure from different soursop preparations. |
| 21 | Ilango S et al. | 2022 | Multiple parts | Alkaloids (coclaurine, reticuline), acetogenins (annomuricin A–C), phenolics, flavonoids | Comprehensive review of anticancer activity highlighting coclaurine, reticuline, and annomuricin among key bioactive compounds | Directly supports the selection of coclaurine (MODERATE risk, CYP2D6), reticuline (MODERATE risk, CYP2D6), and annomuricin (NEGLIGIBLE risk) as representative compounds in our CYP450 analysis. |
| 22 | Al Kazman BSM et al. | 2022 | Leaves, seeds | Alkaloids, acetogenins, flavonoids, coumarins | Comprehensive phytochemical and pharmacological review covering traditional uses, isolated compounds, and biological activities | Key reference for compound identification; documents the breadth of alkaloid and acetogenin diversity in A. muricata that informed our selection of 12 representative compounds across four structural classes. |
| 23 | Mohammed FS et al. | 2024 | Leaves | Essential oils (α-pinene, β-pinene, β-elemene, δ-elemene) | Comprehensive volatile compound profiling of A. muricata leaves using GC-MS, identifying sesquiterpenes and monoterpenes | Recent evidence confirming the presence of sesquiterpene and monoterpene constituents in leaves. Supports the inclusion of germacrene D and (E)-caryophyllene in our analysis, both classified as LOW risk. |
| 24 | Reshma R & Hena M | 2024 | Leaves | Kaempferol-3-O-glucoside, procyanidins, catechin, quercetin, acetogenins | Updated review of phytochemical properties and therapeutic applications identifying both polyphenols and acetogenins as major bioactive classes | Confirms the chemical diversity of leaf preparations; the co-occurrence of flavonoids and acetogenins with alkaloids in crude extracts underscores the importance of multi-compound CYP interaction assessment. |
| 25 | Montejo-Mendez MJ et al. | 2025 | Fruits | Polyphenols (13.10–126.59 mg GAE/gE), flavonoids (73.48–592.70 mg RE/gE) | Genetic analysis and phytochemical profiling of A. muricata fruits demonstrating significant intra-species variation in polyphenol and flavonoid content | Most recent reference documenting substantial quantitative variation between genotypes. Directly supports our limitation statement about regional and genetic variability influencing phytochemical composition and exposure levels. |

This table S1 summarizes the phytochemical literature (2007–2025) that informed the selection of twelve representative compounds for CYP450 interaction analysis in this study. Compound selection was based on: (a) confirmed presence in A. muricata leaves or related tissues, (b) representation of the four major structural classes (aporphine alkaloids, benzylisoquinoline alkaloids, acetogenins, and sesquiterpenes), and (c) reported biological activities relevant to pharmacokinetic interactions. The "Relevance to This Study" column explicitly links each reference to the compounds and risk categories identified through our integrated dual-platform analysis (SuperCYPsPred + SwissADME).

**Abbreviations:** GI, gastrointestinal; BBB, blood-brain barrier; CYP, cytochrome P450; GAE, gallic acid equivalents; RE, rutin equivalents; GC-MS, gas chromatography-mass spectrometry; DPPH, 2,2-diphenyl-1-picrylhydrazyl; ABTS, 2,2′-azino-bis(3-ethylbenzothiazoline-6-sulfonic acid).

***Note:*** *The twelve compounds selected for this study were: anonaine, xylopine, isolaureline (aporphine alkaloids); coclaurine, reticuline (benzylisoquinoline alkaloids); stepharine (proaporphine); swainsonine (indolizidine alkaloid); annonacin, bullatacin, annomuricin (acetogenins); germacrene D, (E)-caryophyllene (sesquiterpenes). SMILES structures were obtained from PubChem (Kim et al., 2023).*

**Table S2.** Predicted CYP interaction profiles, SwissADME CYP inhibition, and representative chemotherapeutic substrates at risk for twelve *A. muricata* compounds. BIQ = benzylisoquinoline.

| **Compound** | **Class** | **Mean Prob. (%)** | **Active CYP Isoforms** | **SwissADME CYP Inhib.** | **Representative Substrates at Risk** |
| --- | --- | --- | --- | --- | --- |
| Isolaureline | Aporphine | 61.5 | CYP1A2, CYP2D6 | 1A2, 2D6 | Tamoxifen, codeine, erlotinib |
| Xylopine | Aporphine | 59.9 | CYP1A2, CYP2D6 | 1A2, 2D6 | Tamoxifen, codeine, erlotinib |
| Anonaine | Aporphine | 55.9 | CYP1A2, CYP2D6 | 1A2, 2D6 | Tamoxifen, codeine, erlotinib |
| Stepharine | Proaporphine | 32.5 | CYP1A2, CYP2C19, CYP2D6, CYP3A4 | 1A2, 2C19, 2D6, 3A4 | Tamoxifen, docetaxel, cyclophosphamide |
| Coclaurine | BIQ | 34.1 | CYP2D6 | 2D6 | Tamoxifen, codeine |
| Reticuline | BIQ | 31.0 | CYP2D6 | 2D6 | Tamoxifen, codeine |
| Germacrene D | Sesquiterpene | 20.1 | CYP2C9 | 2C9 | Warfarin, celecoxib |
| (E)-Caryoph. | Sesquiterpene | 17.9 | None | None | — |
| Annonacin | Acetogenin | 14.1 | CYP3A4 | 3A4 | Docetaxel, irinotecan |
| Bullatacin | Acetogenin | 15.1 | None | None | — |
| Annomuricin | Acetogenin | 14.1 | None | None | — |
| Swainsonine | Indolizidine | 10.7 | None | None | — |
